# Music Emotion Classification Method Based on Deep Learning and Improved Attention Mechanism

**DOI:** 10.1155/2022/5181899

**Published:** 2022-06-20

**Authors:** Xiaoguang Jia

**Affiliations:** School of Music, Baotou Teacheis' College, Inner Mongolia University of Science and Technology, Baotou, Inner Mongolia 014030, China

## Abstract

Since the existing music emotion classification researches focus on the single-modal analysis of audio or lyrics, the correlation among models are neglected, which lead to partial information loss. Therefore, a music emotion classification method based on deep learning and improved attention mechanism is proposed. First, the music lyrics features are extracted by Term Frequency-Inverse Document Frequency (TF-IDF) and Word2vec method, and the term frequency weight vector and word vector are obtained. Then, by using the feature extraction ability of Convolutional Neural Network (CNN) and the ability of Long Short-Term Memory (LSTM) network to process the serialized data, and integrating the matching attention mechanism, an emotion analysis model based on CNN-LSTM is constructed. Finally, the output results of the deep neural network and CNN-LSTM model are fused, and the emotion types are obtained by Softmax classifier. The experimental analysis based on the selected data sets shows that the average classification accuracy of the proposed method is 0.848, which is better than the other comparison methods, and the classification efficiency has been greatly improved.

## 1. Introduction

Due to the complexity of music duration and composition, the emotional features extracted from music show the characteristics of large quantity, multiple dimensions, and difficult to analyze [1]. The results of music emotion classification can be well applied to music recommendation function to reduce the disadvantages of collaborative filtering recommendation [2, 3]. At the same time, music can artistically express the emotional information contained therein, and listeners can also obtain emotional tendency through music audio and lyrics information [4]. Music emotion analysis can be well applied to the music recommendation function. Major online music applications have launched the music recommendation function to recommend suitable music and improve the user experience by analyzing the listening habits of different users [5, 6]. However, most of the applications recommend popular songs but ignore personalized works, which is difficult to meet the needs of listeners under different emotions. Therefore, the research on emotional classification of songs has a good application prospect [7].

Before the emergence of intelligent algorithms, the way of establishing classification labels mainly depended on manual work, and the songs with different music styles were established into corresponding song lists [8]. However, such methods are not only inefficient, but also have high requirements for manual experience, and the classification accuracy is also uneven [9, 10]. On the basis of manual classification, the traditional classification methods are gradually mature, which mainly include methods such as logistic regression, naive Bayes, random forest, and support vector machine [11]. For example, Rao Veeranki et al. [12] analyzed and compared the performances of four traditional methods in the process of music emotion classification: logical regression, naive Bayes, random forest, and support vector machine, and took the parameters such as mean, variance, kurtosis, and skewness as analysis indicators, which effectively improved the efficiency of music emotion classification [12]. However, the targeted feature extraction in mixed audio needs to be improved. Kumar and Vardhan [13] made full use of other emotional features according to the rule-based emotion classification algorithm, and obtained better classification accuracy by adding more words to the dictionary [13]. However, the granularity segmentation of music needs to be further improved. Tiple and Patwardhan [14] proposed a new emotion classification system through link preprocessing, feature extraction, and classification steps [14]. The proposed Spiking Neural Network (SNN) classifier based on gradient descent was used to process frames and extract the time, spectrum, and energy features related to music. Combined with the optimal weight value to reduce the training error, the gradient descent method was optimized. Chen and Li [15] proposed a multi-modal ensemble learning method based on stacking [15]. This method was different from the feature-level and decision-level fusion methods. However, this classification method is inefficient in the environment facing a large number of new music creation, and cannot flexibly meet the needs of category expansion in the later stage [16].

Nowadays, classification algorithms based on machine learning methods and deep neural network learning have carried out extensive research in the fields of audio, image and text, and achieved rich results [17, 18]. With the rise of artificial intelligence-related technologies, computers can realize complex emotion analysis and calculation, and automatically output emotion analysis results through algorithms. Scholars' researches on music emotion feature extraction and classification model are also gradually carried out. Hizlisoy et al. [19] proposed a music emotion recognition method based on CNN-LSTM [19]. The experimental evaluation on the constructed emotional music database effectively showed the good performance of the proposed method. However, this method ignores the timing features of audio itself. Sorussa et al. [20] proposed a digital music emotion classification system with different emotion categories, which used supervised machine learning technology to identify the acoustic features and create prediction models [20]. This method effectively improves the accuracy of the algorithm classification, but the efficiency of machine learning needs to be improved. Gan [21] proposed a recurrent neural network method with channel-attention mechanism to classify the music features [21]. The above machine learning methods have achieved good results in the field of music emotion classification, but in the process of dealing with lyrics and melody, the relationship between lyrics and melody emotions is separated in different ways, without considering the consistency of emotion between lyrics and melody [22], so there is room for further optimization of the detailed classification of music emotion.

Aiming at the problem that most existing classification methods are difficult to deal with multi-dimensional and complex music texts, a music emotion classification method based on deep learning and improved attention mechanism is proposed. Its innovations are summarized as follows:

1. Aiming at the problem of high dimension and sparsity of word vector, the proposed method combines CNN and LSTM to construct emotion classification model, and integrates the matching attention mechanism to further improve the classification accuracy.
2. In order to solve the problem of insufficient performance of single feature classification, the proposed method uses CNN-LSTM model and deep neural network to process word vector and word frequency weight vector, respectively, and carries out feature concatenation to ensure the reliability of emotion classification.

## 2. Lyrics Feature Extraction

### 2.1. TF-IDF Feature Extraction

Term Frequency-Inverse Document Frequency (TF-IDF) is a feature extraction method that represents the weights according to the frequency of word items in the text. TF-IDF can calculate the number of word occurrences by means of probability statistics, evaluate the proportion of word items in the text to determine the importance of the word, and use this to represent the emotional polarity of the lyric text. The more times an emotional representative word appears in a lyrics text, the higher the importance of the emotional word in the emotional classification evaluation. Integrating all word frequency information, the emotional tendency of the whole lyrics text can be evaluated [23].

TF is the word frequency of a word, indicating the number of times a word item appears in the text. TF is calculated as follows:

(1) $$\begin{array}{c} \text{tf}\left(i,j\right)=\frac{n\left(i,j\right)}{\sum_{k}n\left(k,j\right)}, \end{array}$$

where *n*(*i*, *j*) represents the number of times that word *w*_*i*_ appears in document d_*j*_, and its denominator represents the total number of words in the document.

IDF is the inverse text frequency. The fewer times the current text contains word items, the stronger the classification ability of the word items to the text. It can be obtained by dividing the total number of words in the data set by the number of samples containing word items and through logarithmic operation. IDF is calculated as follows:

(2) $$\begin{array}{c} \text{idf}\left(i\right)=\log\frac{\left|D\right|}{\left\{j:t_{i}\in d_{j}\right\}}, \end{array}$$

where |D| represents the number of all documents, and the denominator represents the number of documents containing the word *w*_*i*_.

The TF-IDF calculation result is represented by the product of TF and IDF. If the current word item appears less in the current category and more in the overall sample, the larger the TF-IDF value, the higher the classification ability of the feature item. To sum up, the core of TF-IDF text feature extraction method is to remove the influence of common words and retain important features with text resolution.

TF-IDF is a simple and convenient text word frequency feature extraction method, but it also has some defects. The words in the text are regarded as independent feature items, ignoring the relationship between words and ignoring the relationship between words and the whole article. This representation method has good statistical significance for local content, but ignores the integrity of the text, resulting in the loss of fine-grained emotional semantic content. For example, a certain emotion polar word only appears in the song lyrics text of this emotion, but less in other emotion types, which will lead to the error of emotion evaluation.

### 2.2. Word2vec Word Vector

**Figure 1:**
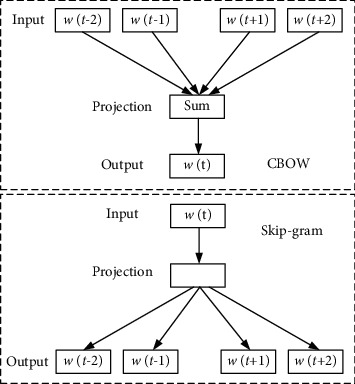
Word2vec training word vector model.

Word2vec is a distributed text representation method, which maps each word item in the text to a word vector. Word2vec improves the shortcomings of the traditional deep learning word embedding model, with faster training speed and fewer vector dimensions. Word2vec usually includes two model structures: Continues Bag of Words (CBOW) and Skip-Gram, as shown in Figure 1.

The model includes input layer, projection layer, and output layer. In CBOW method, the surrounding words are used to predict the central word, so as to use the prediction results of the central word to represent the a priori probability; Skip-Gram uses the central word to predict the surrounding words, so as to predict the overall result and represent a posteriori probability. Therefore, CBOW will be faster than Skip-Gram in practical use. The parameter dimension of Word2vec-generated word vector is related to the number of hidden layer units in the network. The default value of the program is 100 dimensions.

Word2vec also has some defects: because words and vectors are one-to-one, the problem of polysemy cannot be solved; a static word vector representation, although it has strong universality, it cannot be dynamically optimized for specific tasks. For special text types such as lyrics, text information is different from evaluation text, which can be expressed directly through the emotion polar words. The implicit semantic expression in lyrics is often difficult to summarize emotion through separated word frequency information. The word vector method integrating context semantic information has better classification performance [24].

## 3. Proposed Lyrics Emotion Classification Model

### 3.1. Model Construction

**Figure 2:**
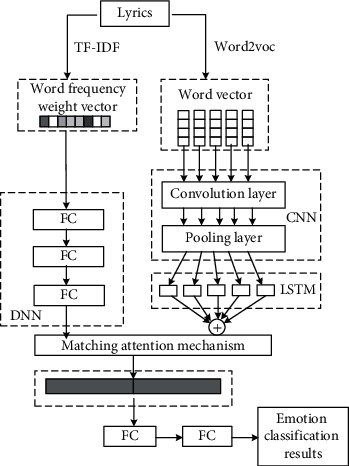
Emotion classification model of lyrics based on CNN-LSTM.

After preprocessing the lyrics text, two emotion feature vectors, vector space model and distributed vector representation, can be extracted. Word2vec is extracted as word vector representation, which can be applied to deep learning methods, but it often has the characteristics of high dimension and sparsity. A single network model cannot deal with the features well. The architecture of CNN-LSTM not only has the advantage of CNN extracting local features, but also has the advantage of LSTM connecting the extracted features in sequences. Although TF-IDF representation method based on word frequency statistics has some defects in the semantic representation, it also has good distinguishing ability for text information with prominent keywords. In order to integrate the emotional feature representation of two kinds of text, a lyric emotion classification model based on CNN-LSTM is constructed. The model architecture is shown in Figure 2.

The proposed model is divided into two parts: word vector + CNN-LSTM and word frequency weight + Deep Neural Network (DNN). First, CNN is used to extract multiple sets of word vector features of the input text, and the extracted features are integrated into the input of LSTM neural network to output a new set of word vector feature representation. Then, the word bag model vector extracted by TF-IDF is processed by DNN. The features of the two categories are concatenated as the fusion representation of lyrics text, which is finally classified and output by Softmax to obtain the text emotion classification results.

The lyrics emotion classification model based on CNN-LSTM is similar to the audio emotion classification model in network structure. The inputs of the audio classification model are spectrogram and low-level descriptor features, respectively, while the inputs of the text classification model are word vector and word frequency weight vector, respectively [25]. The proposed model also has some performance differences when applied to audio features and text features. Because the audio feature dimension depends on the extracted spectrum description feature, the sequence length depends on the segmentation mode and frame interval of the original music; the text feature dimension depends on the distributed representation dimension set in the feature extraction stage, and the length of the text sequence is directly related to the number of word items. For the theme of song audio classification, CNN plays a leading role in the CNN-LSTM combined network. CNN is used to extract spectrum feature, which requires deeper convolution operation. The size of convolution kernel and stride will affect the classification performance. For the propose of lyrics text classification, the original serialized text feature word vector is difficult to train due to its high dimension and sparsity. CNN mainly provides feature compression ability. Bidirectional LSTM and matching attention mechanism have a greater impact on classification accuracy than convolution layer.

**Figure 3:**
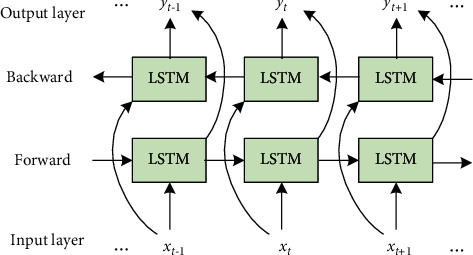
BiLSTM structure.

BiLSTM is a structure composed of forward LSTM and backward LSTM, which can well complete the extraction of data features. BiLSTM can well analyze bidirectional data information and provide more fine-grained calculation. The calculation process is as follows:

(3) $$\begin{array}{c} {\overset{⟶}{h}}_{t}=f\left(\overset{⟶}{W}\cdot x_{t}+\overset{⟶}{W}\cdot {\overset{⟶}{h}}_{t-1}+\overset{⟶}{b}\right),\\ {\overset{\leftarrow }{h}}_{t}=f\left(\overset{\leftarrow }{W}\cdot x_{t}+\overset{\leftarrow }{W}\cdot {\overset{\leftarrow }{h}}_{t-1}+\overset{\leftarrow }{b}\right),\\ y_{t}=g\left(U\cdot \left[\overset{\leftarrow }{h};\overset{⟶}{h}\right]+c\right), \end{array}$$

where, one LSTM layer processes the sequence from left to right, and the other LSTM layer processes the sequence from right to left. $\overset{⟶}{W}$ and $\overset{\leftarrow }{W}$ represent the network hidden layer parameters, *x*_*t*_ represents the input data, ${\overset{\leftarrow }{h}}_{t}$ and ${\overset{⟶}{h}}_{t}$ represent the output results of the two LSTM layers at time *t*, $\overset{⟶}{b}$ and $\overset{\leftarrow }{b}$ represent the offset value, and *y*_*t*_ represents the output of BiLSTM. The BiLSTM structure is shown in Figure 3.

### 3.2. Model Description

#### 3.2.1. Input Layer

The input of the model is audio feature data. The original music file is preprocessed, and the word vector and word frequency weight vector are obtained, respectively. The feature size of each spectrogram is normalized to 256 × 256 × 3, where 256 is the width and height of the image, and 3 represents the number of channels (RGB) of the color spectrogram.

#### 3.2.2. CNN Layer

**Figure 4:**
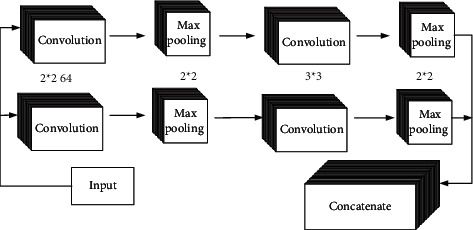
CNN model.

The detailed view of the CNN layer model is shown in Figure 4. In the model implementation, CNN layer includes two convolution layers and two pooling layers. The input of first convolution is the word vector, the convolution operation is performed through 64 convolution kernels with size of 2 × 2 and step of 1, and Relu is used as the activation function [26].

The output vector size *H* of CNN depends on G (input size), *κ* (convolution kernel size), *P* (padding size), and *τ* (step size). The calculation is as follows:

(4) $$\begin{array}{c} H=\frac{\left(G-\kappa +2P\right)}{\tau +1}. \end{array}$$

During the convolution feature extraction, first, use a single convolution kernel to calculate each local feature of the input. Then, concatenate the calculated features vertically, and finally perform nonlinear calculation on the concatenated features through the activation function to obtain the final convolution feature. The mathematical expression is as follows:

(5) $$\begin{array}{c} h_{1\kappa }\left(i\right)=f\left(J_{\kappa }\cdot X\left(i:i+\kappa -1\right)+b\right),\\ \;h_{1\kappa }=\left[h_{1\kappa }\left(1\right);h_{1\kappa }\left(2\right);\cdots ;h_{1\kappa }\left(H\right);\right],\\ hr_{1\kappa }=\text{Relu}\left[h_{1\kappa }\right], \end{array}$$

where, *J*_*κ*_ represents the convolution kernel with height *κ*, H is the size of the output vector, *h*_1*κ*_(*i*) is the *i*-th local feature, *hr*_1*κ*_ is the output c8onvolution feature, *X* is the input, and *f* is the tanh activation function.

#### 3.2.3. LSTM Layer

In order to fuse different features to improve the classification accuracy, cascade is used to connect the merged results as the input of LSTM layer. The mathematical expression is as follows:

(6) $$\begin{array}{c} hp_{1\kappa }=\max\left[hr_{1\kappa }\right],\\ h_{1}=\varphi \left(hp_{1\kappa },hp_{2\kappa }\right), \end{array}$$

where, hp_1*κ*_ is the result of pooling operation; *φ* is the merge connection function, and *h*_1_ is the input of LSTM.

**Figure 5:**
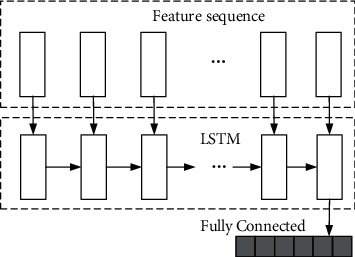
LSTM model.

The word vectors generated from the sample set are further extracted by the CNN layer. Specifically, for the lyrics sample represented as [*v*_(1)_, *v*_(2)_,…, *v*_(*T*)_], where *T* is the number of frames after lyrics segmentation. After passing through the CNN layer, a sequence vector [*c*_(1)_, *c*_(2)_,…, *c*_(*T*)_] is obtained as the input of the LSTM layer. The detailed view of LSTM model is shown in Figure 5.

Input the vector output from the previous layer and selected by the feature into the bidirectional LSTM. The LSTM layer in the model has 128 units, and the output results can be expressed as [*l*_(1)_, *l*_(2)_,…, *l*_(*T*)_].

#### 3.2.4. Matching Attention Mechanism

For fine-grained emotion analysis, the ordinary attention mechanism cannot accurately extract the target words of fine-grained elements, resulting in the low accuracy of emotion analysis. Therefore, based on the original attention mechanism, a matching attention mechanism is built to improve this problem. The input of attention matching mechanism includes two parts. One part is the weighted word vector after the feature fusion of Word2vec word vector feature and TF-IDF feature based on word frequency statistics; the other part is the word vector generated after the fine-grained feature information in the data set is sent to Word2vec. First, these two parts of input are fed into the matching attention mechanism, and the context information and fine-grained element information *q*_*s*_ are captured at the same time. The calculation is as follows:

(7) $$\begin{array}{c} q_{s}=\text{Average}\left(\frac{1}{m}\sum_{i=1}^{m}e_{a_{i}},\frac{1}{n}\sum_{j=1}^{n}e_{\omega_{j}}\right), \end{array}$$

where, Average represents the average value of the input vector, *e*_*a*_*i*__ is the word vector, *e*_*ω*_*j*__ is the weight word vector, and *m* and *n* are the numbers of word vectors and weight word vectors, respectively. In order to make the information of fine-grained elements meaningful, only the dimensions related to fine-grained elements will be retained in the *Q*_*t*_, while other dimensions will be deleted. The calculation process is as follows:

(8) $$\begin{array}{c} Q_{t}=\omega_{t}\cdot q_{s}+b_{t}, \end{array}$$

where *Q*_*t*_ is the weight vector of *k* fine-grained elements. It mainly looks for the dimensions related to fine-grained elements by looking at the words nearby in the word vector space. *ω*_*t*_ and *b*_*t*_ represents the weight matrix and offset vector, respectively. After the loss function is determined, the parameters in *ω*_*t*_ and *b*_*t*_ can be updated by gradient descent method. When the loss function converges, the optimal solution can be obtained. Then multiply *Q*_*t*_ by a randomly initialized matrix *ψ* to obtain the target word *a*_*s*_ matching the fine-grained elements identified by matching attention, which is expressed as follows:

(9) $$\begin{array}{c} a_{s}=\psi \cdot Q_{t}, \end{array}$$

where, the dimension of *ψ* is *k* ×  *d*. It can be updated by gradient descent method.

Finally, matching attention weight *p*_*i*_ is calculated according to *a*_*s*_. The calculation is as follows:

(10) $$\begin{array}{c} p_{i}=\frac{\exp\left(\tanh\left(h_{i}^{T}\cdot \omega_{0}\cdot a_{s}\right)\right)}{\sum_{j=1}^{n}\exp\left(\tanh\left(h_{j}^{T}\cdot \omega_{0}\cdot a_{s}\right)\right)}, \end{array}$$

where *ω*_0_ ∈ *ℜ*^*d*×*d*^ is a trainable weight matrix, *h* is the output of LSTM hidden layer.

Finally, the weighted sum of the hidden vector *h*_*i*_ generated by the bidirectional LSTM and the matching attention weight *p*_*i*_ is used for the sentence representation *Z*_*s*_ of emotion classification, which is expressed as follows:

(11) $$\begin{array}{c} Z_{s}=\sum_{i=1}^{n}p_{i}h_{i}. \end{array}$$

Take *Z*_*s*_ as the feature of the final emotion classification and put it into the fully connected layer for linear transformation, and Softmax classifier is used for emotion classification to obtain the final emotion *ϕ*. The mathematical expression is as follows:

(12) $$\begin{array}{c} \phi =\text{softmax}\left(\omega_{Z}\cdot Z_{s}+b_{Z}\right). \end{array}$$

#### 3.2.5. DNN Layer

The input of DNN layer is the word frequency weight vector, which contains three hidden layers, also known as FC (fully connected layer). All nodes of FC in the network are connected with the nodes of the previous layer to achieve the purpose of integrating feature information and reducing dimension. The number of nodes of the three FCs in the model is 256, 128, and 64, respectively. The dimension of the input feature is further compressed after passing through the DNN layer.

#### 3.2.6. Output Layer

The output layer consists of FC and Softmax. First, the output of word vector features through CNN layer, LSTM layer, and attention mechanism layer and the output of word frequency weight vector features through DNN layer are concatenated as the final classification feature vector representation. Output classification is through FC and Softmax layers. The Softmax layer is a loss function, which is used to map the output to the probability interval to obtain the classification probability distribution, so as to output the classification results. The model is actually classified into four emotional categories: happy, sad, healing, and calm.

## 4. Experiments and Analysis

In order to build a parallel corpus of Chinese audio and lyrics, the data source is locked on the domestic music platform, and the data is collected based on the target of the task. In order to select the songs with higher quality, the songs with more credibility are selected, that is, the songs with a playback times of more than 2.5 million. In order to further carry out the task of music emotion classification, four kinds of emotion labels with happy, sad, healing, and calm were selected as candidates. A total of about 6000 music were collected. After further screening of song length, audio quality and language, 5286 music were finally retained as the candidate data set. On this data set, it is divided into two parts: training set and test set. The specific information of each data set is shown in Table 1.

In the experiment, the lyric text is preprocessed. First, the word segmentation is carried out to remove the invalid information related to music composition and stop words, and constructs a pure lyric text word item combination representation. Then, the text is transformed into a digital vector recognized by the computer through different feature extraction methods, and the feature dimension of the text vector is set to 100 dimensions. Finally, input them to the classifier to output the emotion classification results. The parameters of LSTM model are shown in Table 2.

### 4.1. Classification Accuracy of Different Text Features

The experiment adopts different word frequency weight vector feature extraction methods to verify the emotional classification performance of lyrics. First, the preprocessed text is represented by text vector through TF-IDF and Word2vec feature extraction methods, and then the same SVM classifier is used to output the emotion classification results. The classification accuracy of different text extraction methods is shown in Table 3.

As can be seen from Table 3, TF-IDF is completely based on the features of word frequency. When facing the sample data with implicit emotional semantics such as lyrics, the emotional classification performance is slightly insufficient, and the average classification accuracy is only 0.701. At the same time, the distributed word vector feature representation extracted by Word2vec tool has also achieved good accuracy in SVM classifier. The classification accuracy of happy emotion is as high as 0.801 and the average classification accuracy is 0.746. This distributed vector can be well used as the input of deep network method.

### 4.2. Classification Accuracy of Different Attention Mechanisms

In order to study whether the matching attention mechanism can further improve the performance, it is compared with the traditional attention mechanism. The evaluation results of the joint training model on the selected data set are shown in Table 4, in which the average classification accuracy is used for performance evaluation.

It can be seen from Table 4 that the matching attention mechanism significantly improves the classification performance of the model, and its average classification accuracy is 0.826, which is 0.073 higher than that of the traditional attention mechanism. The traditional attention mechanism cannot accurately extract the target words of fine-grained elements, resulting in the low accuracy of emotion analysis. The matching attention mechanism can solve this problem, and greatly improve the classification accuracy combined with the context information.

### 4.3. Experimental Comparison and Analysis with Other Methods

**Figure 6:**
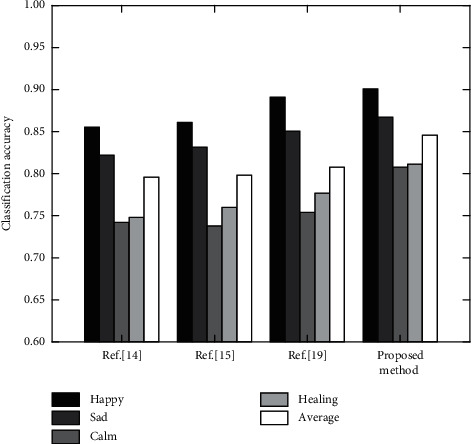
Classification accuracy of different methods.

In order to demonstrate the performance of the proposed method, it is experimentally analyzed with Reference [14, 15, 19] on the selected data set. The classification accuracy of different emotions in lyrics is shown in Figure 6 and Table 5.

As can be seen from Figure 5 and Table 5, the proposed method combines the characteristics of CNN and LSTM, constructs an emotion analysis model based on CNN-LSTM, and combines DNN network learning to greatly improve the accuracy of music emotion classification, with an average classification accuracy of 0.848. The fusion processing of deep learning network improves the classification performance, especially integrates the matching attention mechanism, accurately extracts the target words of fine-grained elements, and significantly improves the classification accuracy of emotional types such as calm and healing, which are 0.056 and 0.045 higher than those in reference [19]. Reference [19] uses CNN-LSTM architecture to complete music emotion recognition. Although the average classification accuracy reaches 0.815, it is easy to confuse emotion types such as calm and healing, and the performance needs to be improved. Reference [14] classifies emotions based on gradient descent SNN classifier, and reference [15] classifies emotions based on stacking multi-modal ensemble learning method. Both of them are difficult to accurately distinguish massive and complex music types, and the average classification accuracy is about 0.800. In conclusion, the proposed method uses the comprehensive feature extraction ability of CNN and the ability of LSTM to process the serialized data to obtain better classification results, and has stable performance and high robustness under each subclassification.

## 5. Conclusion

Music contains rich human emotional information. The study of music emotional classification is helpful to organize and retrieve massive music data. Because of the large number and multiple dimensions of music, it is difficult and incomplete to extract emotional features. Therefore, a music emotion classification method based on deep learning and improved attention mechanism is proposed. The word frequency weight vector obtained based on TF-IDF is input into DNN for feature analysis, and the word vector obtained by Word2vec method is sent into the emotion analysis model based on CNN-LSTM. After the output of the two feature extraction channels are fused, the output layer outputs the emotion type. The experimental results based on the selected data set show that matching attention mechanism can more accurately extract the target words of fine-grained elements and improve the classification performance. Compared with the traditional attention mechanism, its average classification accuracy is improved by 0.073.

The processing of audio features in this paper is still a little rough. Only using the existing common features cannot fully reflect the relationship between music structured information and human emotion. Therefore, the feature extraction method with more music emotion classification ability can be further explored.

## Figures and Tables

**Table 1:** The size of the data set and the number of songs contained in each type of emotion.

	Training set	Test set	Total
Happy	1078	194	1272
Sad	1267	317	1584
Calm	882	221	1103
Healing	1061	266	1327
Total	4288	998	5286

**Table 2:** Parameter setting of LSTM network model.

Parameter	Value
Loss function	Softmax
Optimizer	Adam
Learning rate	0.01
Activation function	Tanh
Dropout	0.03
Batch size	50
Epoch	30

**Table 3:** Classification accuracy results of different text features.

Text features	TF-IDF	Word2vec
Classification method	SVM	
Happy	0.762	0.801
Sad	0.735	0.779
Calm	0.647	0.698
Healing	0.661	0.703
Average	0.701	0.746

**Table 4:** Experimental results of three attention mechanisms.

Model	Classification accuracy
Traditional attention mechanism	0.753
Matching attention mechanism	0.826

**Table 5:** Classification results of different methods.

Method	Reference [14]	Reference [15]	Reference [19]	Proposed method
Happy	0.853	0.861	0.887	0.903
Sad	0.828	0.837	0.849	0.864
Calm	0.742	0.739	0.753	0.809
Healing	0.751	0.766	0.771	0.816
Average	0.794	0.801	0.815	0.848

## Data Availability

The data used to support the findings of this study are included within the article.
